# Differential behavioural and endocrine responses of common voles (*Microtus arvalis*) to nest predators and resource competitors

**DOI:** 10.1186/1472-6785-13-33

**Published:** 2013-09-08

**Authors:** Monique Liesenjohann, Thilo Liesenjohann, Rupert Palme, Jana Anja Eccard

**Affiliations:** 1 Department of Animal Ecology, University of Potsdam, Maulbeerallee 1, D-14469 Potsdam, Germany; 2 Department of Natural Sciences – Biochemistry, University of Veterinary Medicine, Veterinaer-Platz 1, A-1210 Vienna, Austria

**Keywords:** Behavioural adaptations, Small mammals, Interspecific interactions, Nest predation, Stress response, Faecal corticosterone metabolites, Burrow system, Shrews, Voles

## Abstract

**Background:**

Adaptive behavioural strategies promoting co-occurrence of competing species are known to result from a sympatric evolutionary past. Strategies should be different for indirect resource competition (exploitation, e.g., foraging and avoidance behaviour) than for direct interspecific interference (e.g., aggression, vigilance, and nest guarding). We studied the effects of resource competition and nest predation in sympatric small mammal species using semi-fossorial voles and shrews, which prey on vole offspring during their sensitive nestling phase. Experiments were conducted in caged outdoor enclosures. Focus common vole mothers (*Microtus arvalis*) were either caged with a greater white-toothed shrew (*Crocidura russula*) as a potential nest predator, with an herbivorous field vole (*Microtus agrestis*) as a heterospecific resource competitor, or with a conspecific resource competitor.

**Results:**

We studied behavioural adaptations of vole mothers during pregnancy, parturition, and early lactation, specifically modifications of the burrow architecture and activity at burrow entrances. Further, we measured pre- and postpartum faecal corticosterone metabolites (FCMs) of mothers to test for elevated stress hormone levels. Only in the presence of the nest predator were prepartum FCMs elevated, but we found no loss of vole nestlings and no differences in nestling body weight in the presence of the nest predator or the heterospecific resource competitor. Although the presence of both the shrew and the field vole induced prepartum modifications to the burrow architecture, only nest predators caused an increase in vigilance time at burrow entrances during the sensitive nestling phase.

**Conclusion:**

Voles displayed an adequate behavioural response for both resource competitors and nest predators. They modified burrow architecture to improve nest guarding and increased their vigilance at burrow entrances to enhance offspring survival chances. Our study revealed differential behavioural adaptations to resource competitors and nest predators.

## Background

Communities are shaped by interspecific interactions and specific adaptations, depending on the nature of the interaction (direct or indirect), allowing coexistence [1]. Effects of competition might be masked by behavioural adaptations as a result of a shared evolutionary history. Studies of interspecific competition have mainly focused on how indirect exploitation competition affect behaviour, e.g., via territoriality and foraging strategy [2], and have neglected the implications of direct interference for community structure [3] and evolution [4].

Direct antagonistic interactions in competition for food, space, nest sites, or mating partners can be very costly, especially if the competition reduces individual fitness (direct interference) [3,5-7]. Aggressive behavioural confrontations between competing species are likely if adults prey on each others offspring [8]. Interference may result from resource-related aggression between heterospecific or conspecific individuals with potential effects on niche use and community structure (e.g., dominance or exclusion) [4]. Nest predation has been considered a special case of direct interference via interspecific killing [3,9]. It is a significant source of offspring mortality and can strongly affect parental life history strategies [10,11].

Although numerous population studies have shown that nest predation is widespread in animal communities [12-14], its importance as an evolutionary force has been neglected [15,16]. Recent studies on avian breeding biology demonstrate that the direct fitness effects of nest predation can drive evolution [10,12,17,18]. Safety of nest sites and optimised breeding conditions are key intrinsic components of breeding habitat quality [15,19]. Adults should adapt their behaviour to predation risk [18,20] by, e.g., choosing concealed nest sites [19], adjusting clutch size or nestling period [12], and temporally partitioning foraging activity [21,22].

Comprehensive knowledge about adaptive strategies against nest predation in mammals is lacking. Yet defence of a nest and its vulnerable young is a powerful explanation for the ultimate function of female aggression and territoriality, which is most intense during lactation and near the nest site [23]. In addition, nests (in birds) or burrows (in mammals) serve as micro-refuges from predators and therefore have anti-predatory benefits for the adult as well [24].

### Parental anti-nest predatory behaviour

According to parental investment theory, parents must trade off the benefits of investment into current offspring with possible negative effects on their future inclusive fitness [13,25-27]. In addition, parents might suffer extra costs of defending offspring against predators, including time, energy, and missed opportunity costs [26,28,29]. Short-term adjustments are often trade-offs between behaviours, e.g., predator avoidance and foraging [30], and escalate in defending offspring against predators or infanticidal conspecifics [8,23,26,31,32]. The degree of escalation is influenced by offspring vulnerability and reproductive value to the parent [13,25]. Female rodents become more aggressive during their pregnancy [33]. Female European rabbits (*Oryctolagus cuniculus*) increase vigilance during late pregnancy to minimise predation [34]. In mice, mothers successfully defend their nestling pups against infanticidal conspecifics [32]. Attacks on pups decrease rapidly after they reach a less vulnerable stage [35]. Maternal defence behaviour against rattlesnakes declines in California ground squirrels as snake activity declines when pups grow older [31].

According to the “harm to offspring hypothesis” of Dale et al. [36], the level of parental predation risk-taking is adjusted according to the harm the offspring will suffer without parental care. Most vulnerable are altricial nestlings incapable of escaping and defending themselves [13,36,37]. Predation risk is highest when the parents are away (e.g., during foraging trips) [32,33,37,38]. In response, parents might alter their foraging time (e.g., reducing activity) or seek temporal or spatial foraging refuges to decrease risk [39,40]. Cresswell [41] showed that nesting blackbirds altered their nest defence behaviour to compensate for predation risk demonstrating that parental behavioural flexibility in response to predation can yield fitness benefits [42] compared with fixed strategies [18]. For semi-fossorial mammals, the morphology and complexity of burrows have high defence value for nestlings and thereby fulfil an anti-predator function, especially for animals with helpless altricial young [24,43]. Hunting intruders might be confused by very complex burrow systems [44,45]. Some species dig special parturition chambers in addition to the main burrow (e.g., European rabbits, [46]) and plug them to minimise predation (e.g., Columbian ground squirrel, *Urocitellus columbianus*, [31]). Increased vigilance (e.g., high scanning rates in European rabbits [47,48]) at burrow entrances and adjusting burrow attributes (diameters, depths, or lengths of the burrow, tunnels, and nest chambers) may further lower the risk of nest predation [17,45,49].

### Physiological stress responses to nest predation risk

Physiological responses in stressful situations, e.g., encounters of prey with predators, are evolutionarily conserved and represent a widespread and fundamental mechanism for ecosystem functioning in animal systems [40,50]. Predator-induced changes in stress hormone metabolites associated with acute or chronic risks (reviewed in [40]) aim to increase survival [51]. Animals without adequate alternative defence responses (e.g., alteration of life histories, defensive morphologies) to mitigate predation risk must engage in costly physiological responses [40]. Elevated plasma glucocorticoids, or their faecal metabolites, are often measured as indicators of such physiological stress responses [50,52-55].

### Study system and hypotheses

Here we compare behavioural strategies among different interaction types: interspecific resource competition, intraspecific interference competition, and interspecific nest predation. We used a small mammal study system including the semi-fossorial common voles (*Microtus arvalis*) as focus animals, the greater white-toothed shrews (*Crocidura russula*) as potential nest predators, and field voles (*Microtus agrestis*) as interspecific competitors. All three species coexist in many habitats of the northern hemisphere, overlapping in their common habitat and, to a smaller extent, in their diets [56-58]. Voles live in large burrow systems with underground tunnels and corresponding runway systems above ground. Shrews explore vole tunnel systems to search for invertebrate food [59]. Because of the limited space underground, shrews may react aggressively when encountering tunnel inhabitants. Additionally, they can act as nest predators on altricial vole nestlings by plundering their easily accessible nest chambers [60]. This shrew behaviour might intensify during environmentally-adverse seasons when invertebrate food is scarce, e.g., in autumn [61].

To protect vulnerable altricial nestlings from nest predators, voles should have evolved anti-nest predator strategies to secure their reproductive success. Getz *et al.*[38] reported nest defences in two American vole species, *Microtus pennsylvanicus* and *M. ochrogaster*, even against larger shrew species, e.g., *Blarina brevicauda*. Shrews only successfully preyed on nests when vole mothers were absent on foraging trips [38].

What kinds of parental behavioural strategies can secure nestling survival and allow the coexistence of competing species in a nest predator–prey system? How do these strategies differ from responses to non-nest predator antagonists?

(A) We hypothesised that vole mothers adjust their burrow systems in response to antagonists [45]. If she encounters a nest predator during pregnancy, we expected to find different nest architectures at parturition than in the absence of predators.

(B) We further hypothesised that vole mothers react to nest predation risk by altering their time budgets and habitat use. To guard vulnerable nestlings, vole mothers should increase vigilance and nest guarding behaviour (e.g., at burrow entrances), especially during the first sensitive nestling phase. We expected guarding behaviour to be more intense in the presence of a potential nest predator than in the presence of an inter- or intraspecific resource competitor. In addition, if guarding behaviour incurs extra costs for vole mothers, e.g., owing to shortened foraging periods, we expected that to be reflected in the mother’s or nestlings’ body conditions.

If vole mothers exhibit physiological stress reactions concomitant to these behavioural reactions (A and B), we expected levels of faecal corticosterone metabolites (FCM) to correlate to the presence or absence of different antagonists.

(C) Thus, we hypothesised that the levels of stress hormone metabolites in expectant vole mothers increased when they encountered shrews as nest predators.

## Results

### Behavioural adaptations: burrow architecture

**Figure 1:**
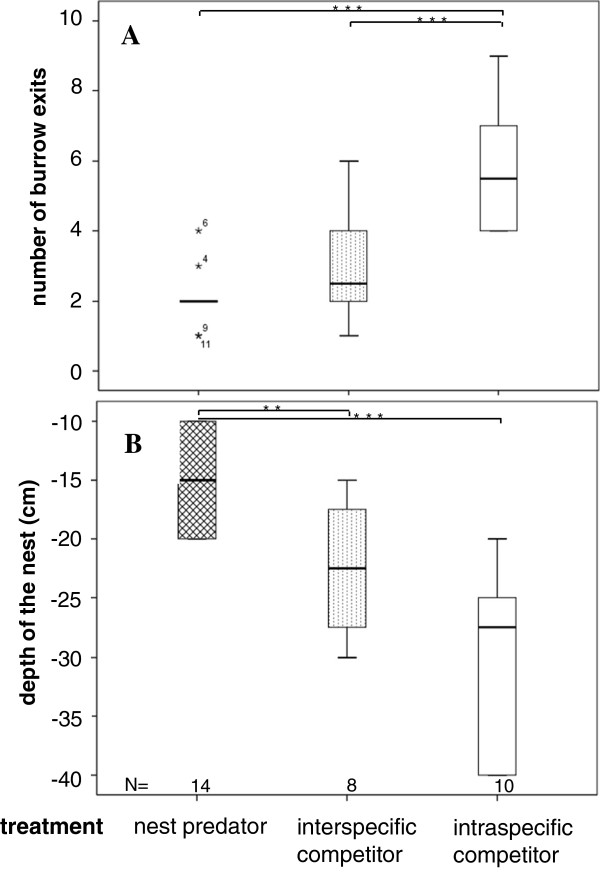
**Number of the burrow entrances (A) and the depth of the nest (B) of vole females (*Microtus arvalis*) in the presence of a nest predator (NP, Greater white toothed shrew, *Crocidura russula*), or a interspecific resource competitor (RC, female field vole, *M. agrestis*) or a intraspecific competitor control (C, female common vole, *M. arvalis*).** All burrow entrances were counted at day 22 of the experiment when vole nestlings were 3 days old. Please note that the y axis in **(B)** is negatively scaled (0 = ground level), visualizing the direction of digging into the ground.

The numbers of burrow entrances built by vole mothers were significantly influenced by the type of antagonist (GLM, *F*_2, 30_ = 16.05, *P* < 0.001; Figure 1A). In the NP treatment, they built fewer entrances (2.1 ± 0.19, mean ± SE) compared with the RC (3.0 ± 0.58) or C (5.8 ± 0.56) treatments. The depth of the nest site was also influenced by antagonist type (ANOVA, *F*_2, 29_ = 18.09, *P* < 0.001; Figure 1B). Mothers built nests closer to the surface in the NP (14.6 ± 1.1 cm) than in the RC (22.5 ± 2.3 cm) or C (29.5 ± 1.9 cm) treatments.

### Behavioural adaptations: activity in burrow entrances

**Figure 2:**
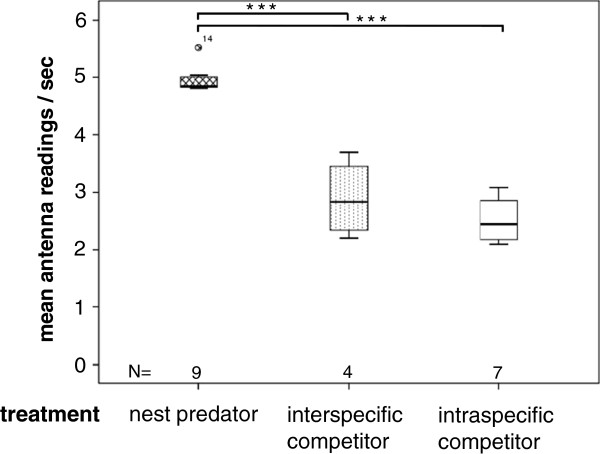
**Vole mother’s (*Microtus arvalis*) time spent in their burrow entrances (during 36 hours).** This variable was measured as mean number of antenna readings per second (possible maximum = 6 readings per second, indicating that the animal sits in the antenna ring; < 3 readings indicate a fast passage) in the presence of a nest predator (NP, shrew, *Crocidura russula*), a interspecific resource competitor (RC, field vole, *M. agrestis*), or a intraspecific competitor control individual (C). Data derived from circular antennas fixed at each burrow entrance connected to automatic transponder reading stations.

The treatment species had a significant effect on the mothers’ activity in the burrow entrances (MANOVA, Wilks’ lambda = 0.079, *F*_6, 30_ = 12,84, *P* < 0.001). Vole mothers in the NP treatment spent more time in entrances (4.9 ± 0.2 antenna readings per second) compared with those in the RC (2.9 ± 0.7 readings/sec) or C (2.5 ± 0.4 readings/sec) treatments (ANOVA, *F*_2, 17_ = 80.1, *P* < 0.001; Figure 2). The mean number of readings/bout (ANOVA, *F*_2, 17_ = 0.863, *P* = 0.44) and the mean number of seconds vole mothers spent in entrances per bout (ANOVA, *F*_2, 17_ = 2.14, *P* = 0.15) were not influenced by the antagonist species.

We also recorded all treatment species entering the tunnel entrances (Table 1). Seventy-eight per cent of the shrews (NP) entered the burrow systems, while only 50% of the field voles (RC) and 43% of common voles (C) did so, but the latter two species visited the burrow system more often per replicate than did the shrews (Table 1).

**Table 1:** Summary of antagonist species visits to vole mothers’ burrow systems

**Treatment animal**	**Replicates in total**	**Number of replicates with visits of treatment individuals**	**Mean visits per replicate**
Nest predator	9	7	1.5
Interspecific resource competitor	4	2	2.0
Intraspecific control	7	3	2.0

The number of antagonist visits to the vole burrow was recorded using automatic transponder-reading antennas inserted into tunnel entrances.

### Stress hormone level

**Figure 3:**
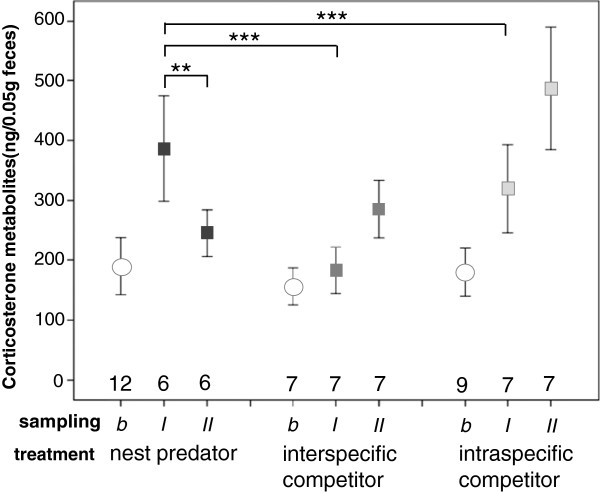
**Amount of corticosterone metabolites in the fecal samples of common vole mothers (*Microtus arvalis*).** Mothers were either treated with a nest predator (NP, shrew, *Crocidura russula*), an interspecific resource competitor (RC, field vole, *M. agrestis*) or an intraspecific competitor control individual (C). Samples were taken at three reproductive phases (sampling *b, I, II*) within one reproduction cycle of each female common vole. *b*: baseline; taken after 3 days of habituation to enclosures (at day 15 of common voles’ pregnancy) before treatment. *I*: Sample taken around parturition and 3 days after adding treatment animals to enclosures (at day 18 of pregnancy). *II*: Sample taken with having 5 days old nestlings.

We found no difference in the baseline amount of FCMs before the treatments (GLM, *F* = 0.138, *df* = 2, *P* = 0.872; Figure 3). Under treatment conditions (sampling *I* and *II*), FCMs were affected by an interaction of treatment and sampling (GLM, treatment × sampling: *F* = 7.636, *df* = 2, *P* = 0.002; treatment: *F* = 4.262, *df* = 2, *P* = 0.023; sampling: *F* = 1.106, *df* = 1, *P* = 0.301; Figure 3). Mothers in NP treatments had a higher FCM levels around parturition (sampling *I*) compared with the other treatments (Bonferroni corrected post hoc comparisons, NP *I* / RC *I*: *P* = 0.001, NP *I* / C *I*: *P* = 0.009) and compared with the postpartum period (sampling *II*) (NP *I* / NP *II*: *P* = 0.014). The FCM of vole mothers did not differ any more between treatments when nestlings were 5 d old (sampling *II*). Non-significant post hoc results are not displayed.

### Maternal body condition and offspring survival

We found no influence of treatment on the vole mother’s body weight (MANOVA, Wilks’ lambda = 0.899, *F*_6, 56_ = 0.51, *P* = 0.79), either prepartum (ANOVA, *F*_2, 30_ = 0.39, *P* = 0.68) nor postpartum (ANOVA, *F*_2, 30_ = 0.03, *P* = 0.97). We also found no influence of antagonist species on offspring survival (MANOVA, Wilks’ lambda = 0.959, *F*_4, 48_ = 0.255, *P* = 0.91). Both the number of vole nestlings (ANOVA, *F*_2, 25_ = 0.193, *P* = 0.83) and nestling body weight (ANOVA, *F*_2, 25_ = 0.371, *P* = 0.69) were independent of antagonist species.

## Discussion

We investigated behavioural and physiological responses of vole mothers to different intra-nest interactions: nest predation, interspecific resource competition, and intraspecific competition. Nest predators affected burrowing behaviour and passage activity at burrow entrances as well as physiological stress responses. The presence of neither a potential nest predator nor a competitor species altered reproductive success. Furthermore, nestlings in all treatments were in good condition. These results indicate that vole mothers seem to perceive shrews as a threat and adequately respond to their presence. Treatment animals of all antagonist species visited the vole mothers’ burrow system several times. Vole mothers were apparently successful in defending their nestlings against potential nest predation attempts.

### Adaptive anti-nest predatory strategies

Pregnant common voles responded to the presence of a potential nest predator or a resource competitor with different nest site preparations. Contrary to findings on African gerbil (*Tatera brantsii*[44]) and prairie vole (*Microtus ochrogaster*[45]) mothers, which build complex burrow systems to confuse predators or to hide nestlings [44], the common voles in our study constructed simpler burrow systems in the presence of nest predators, but not when a competitor species was present. Nest defence might be more efficient in a basic burrow system than in a complex one. Additionally, simpler refuges might help to reduce interactions with antagonists and avoid unnecessary energetic costs [26,28], as in complex tunnel systems. Another explanation might be that pregnant females without nest predation pressure stayed in their prepartum burrow, while pregnant females threatened with a nest predator may have dug a new, isolated nest site [45], as shown in laboratory rats [62] and European rabbits [46]. By preparing the nest site prepartum (e.g., by plugging nest burrow entrances [63]), mothers might try to avoid spending energy postpartum in digging and guarding the nestlings. These findings agree with the “harm to offspring” hypothesis [13,33,36,37], which predicts that mothers should minimise periods of vulnerability during parental absence (e.g., foraging, movement activities, and digging) [33]. Common vole burrow traits in the presence of a heterospecific competitor might reflect a general necessity for nestling protection against possible intruders. Harper and Batzli [45] found no strong effect on the burrowing behaviour of prairie voles by the presence of heterospecific meadow voles. Although heterospecific resource-related aggression can have similar consequences to predation effects (e.g., competitor exclusion) [4], vole mothers seemed to differentiate between the risks of heterospecific aggression and nest predation.

Nest guarding and vigilance behaviour as indicators of the perceived predation risk suggest that vole mothers have adapted to shrews as nest predators through a shared evolutionary past. Only in the presence of shrews did vole mothers spent long time periods in the burrow entrances. Increase of vigilance is a common response to predator odour [48,64,65]. For example, female European rabbits increased their vigilance (scanning rates), especially during late pregnancy, to deter attacks by potential predators [34].

Female mammals must allocate their time among maternal care, foraging, and vigilance, especially during the first days of nestling life. In some species, this trade-off adversely affects the condition of pregnant or lactating females [66-68]. We found no effect of increased vigilance in the shrew treatment on neither maternal nor nestling body weight. Other studies with simulated predation risks also demonstrated stable food intake, despite increased vigilance [34,48,69]. Vigilance and foraging during the energetically-demanding reproductive phase are therefore not mutually exclusive, and mothers in our experiment were able to successfully protect their young against nest predators without suffering energetic costs. This finding might not apply under natural conditions, e.g., if food for shrews is scarce and shrews exert higher predation pressure on vole nestlings.

### Physiological stress responses to nest predation

The physiological stress response to the presence of a nest predator during late pregnancy was higher than in other competitor treatments or in the postpartum phase. A few days after parturition, the levels of FCMs decreased almost to pre-treatment baseline levels. Female rodents were most aggressive around parturition, and aggression levels decreased thereafter [33]. Pregnant laboratory rats, for example, more aggressively defend their nests against conspecific males and females [62]. Our results probably indicate a correlation between aggression and stress hormone levels and suggest that hormonal-mediated adaptive behavioural strategies help to minimise the risk of nest predation.

Vole mothers in the presence of a heterospecific or conspecific competitor had higher FCM levels during the postpartum phase compared with the nest predator treatment and the prepartum phase. Increased resource competition (‘exploitative competition’) during lactation associated with higher aggression levels against competitors [4] might have caused the elevated FCM levels.

## Conclusions

Vole mothers in our study showed behavioural and hormonal reactions specific to antagonist species. The presence of a nest predator induced flexible behavioural adaptations during pregnancy to secure subsequent offspring survival, including modifications of the burrow architecture in combination with increased vigilance and nest guarding. Behavioural adaptations seem to be successful maternal strategies to balance typical postpartum parental investment trade-offs (e.g., time for foraging and nest defence) and to secure fitness. Our results suggested differential adaptations as a result of co-evolution with a nest predator compared with resource competitors. Voles and shrews do not only compete in a ‘race for space’ (interference competition, [61]) but also in an ‘arms race’ in predatory and anti-predatory behaviour.

## Methods

### Study site

We conducted the experiment in eight caged outdoor enclosures (5 × 6 m) at the Department of Animal Behaviour at the University Bielefeld, Germany (52°02’N, 8°29’E). Enclosures were caged with micromesh to prevent avian and ground predation and equipped with multiple-capture live traps (six per each enclosure; Ugglan, Grahnab, Gnosjö, Sweden). The ground was densely covered with grassland herbs.

### Experimental schedule and animals

Female common voles were used as focus animals and observed during late pregnancy, parturition, and early lactation. The common voles were wild-caught or laboratory-born multiparous individuals and each was used only once during the experiment. Age group and origin of both focus and treatment animals were equally distributed over experimental treatments and replicates. We used different antagonists as treatment animals: greater white toothed shrews were potential nest predators (NP), non-pregnant female field voles were interspecific resource competitors (RC), and non-pregnant female common voles were intraspecific resource competitors (control, C). The study design involved a constant competitor density to allow comparisons among competitor types rather than whether shrews had an effect at all (for a discussion of additive or replacement designs see [1,70])*.* We expected vole females with a conspecific female to behave similarly to being alone because common voles are very sociable and not solitary or exclusively territorial.

Seven experimental runs with parallel enclosure replicates were carried out between June and September 2008, the main reproductive phase of the common vole. There were 32 realised enclosure replicates in total, with one focus female each. One run consisted of at least two parallel enclosure replicates including one nest predator and one resource competitor treatment (NP, 14 replicates; RC, 8 replicates; and C, 10 replicates).

All focus and treatment animals were individually marked with passive integrated transponders (PIT ID100; Trovan^®^). Because shrews are very sensitive to trapping stress, we checked mealworm-baited traps every hour. Common voles, field voles, and shrews were caged separately to prevent odour exchange prior to the experiment. Shrews were fed mealworms, house crickets, hand-trapped spiders, and bugs. Both vole species were fed standard mouse laboratory food. We used both sexes of the shrews for our experiment because they were difficult to trap; sexes were distributed equally over runs.

Adult common voles were paired in the laboratory at the beginning of each run (Table 2) and usually mated immediately after pairing. Pregnant females were released into enclosures for habituation 12 d after pairing. After 3 d of habituation, we sampled the faeces of the focus females to get a stress hormone baseline (*b*, Table 2) on a natural grass diet. To minimise the influence of trapping stress, we checked traps every hour.

**Table 2:** Schedule of experimental steps and faecal sampling during each experimental run

**Experimental day**	**Experimental steps**	**Faecal sampling**
**0**	voles paired in laboratory	
**3**	males removed	
**12**	pregnant females released into enclosures for habituation	
**15**	treatment animals (NP, RC, C) added to enclosures	*b*: baseline, prior to the experiment
**18**	females equipped with radio tags	*I*: sample under treatment conditions, prior to parturition
**19-21**	parturition, nest site identification by radio telemetry	
**22-24**	detection of nest entrances by fogging, activity measurements by automatic transponder reading antennas	
**24**	burrow characteristics and offspring parameter	*II*: sample under treatment, with nestlings

Each trapped female was placed into a sample chamber (diameter: 130 mm, height: 100 mm) with a metal bottom with holes (5 × 5 cm) for a maximum of 2 h to get faecal samples. All excreta fell onto filter paper and were collected into an Eppendorf tube. We sampled the pre-trapping stress levels in faeces collected within 2 h of trapping to avoid the confounding effects of trapping stress itself, which appears in faeces after 3–4 h [71]. In common voles, the stress of retrieving and handling a caged animal produces a hormone metabolite peak in the faeces after 2 h (unpublished data). Tubes with faeces were immediately frozen at -80°C.

We added one treatment animal to each enclosure. In four cases (one NP, two RC, and one C), we had to stop the replicate, because the focus female had apparently terminated the pregnancy. In NP treatment enclosures, we placed a food station in the middle of the enclosure to provide mealworms and water for the shrew. A filter paper beneath each feeding station was surrounded by a permanent ink pad and used to check for footprints daily to confirm shrew presence.

On day 18 of pregnancy (3 d after adding the treatment animal), we collected a faecal sample from each focus female to determine the stress hormone level around parturition (*I*, Table 2). Females were equipped with radio telemetry tags (Biotrack, Wareham, UK) to locate their nest sites during parturition (days 19–21 of pregnancy).

After parturition, we counted burrow entrances using a fogging machine (N-110; Eurolite) with an odourless, biodegradable, non-irritant, and non-toxic fogging fluid (Fogging Fluid B; Eurolite). To monitor the presence of pitted animals (shrews and voles) at burrow entrances, we inserted circular antennas (type EUR 3120, diameter: 5 cm, EURO I.D., Weilerswist, Germany) into each tunnel entrance (circa 5 cm deep) and measured passage activities for 3 d. Antennas were connected to automatic radio-frequency identification transponder reading stations (type LID 665, EURO I.D.) powered in the field by a 12 V lead battery with a 5 m long cable.

On day 24 of the experimental run, we collected a faecal sample to estimate stress hormone levels of mothers with nestlings in the presence of treatment animals (*II*, Table 2). While mothers were in the sampling chamber, we excavated the nests and recorded the depth of the nest chamber, the number of entrances, and the number and weight of nestlings. All nests were recovered with healthy, living nestlings that re-joined their mother after the replicate. After the experiment, all treatment animals were released back to their capture site.

### Measurement of faecal corticosterone metabolites

FCMs were extracted as described by Touma *et al.*[72] and analysed using 5α-pregnane-3ß,11ß,21-triol-20-one enzyme immunoassays (for details see [73]). This method was validated for common voles prior to this study (Liesenjohann in prep.) following Touma *et al.*[72] for mice.

### Analyses and statistics

To allow the animals to habituate, we began data acquisition 24 h after inserting the antennas into the tunnel entrances. Activity bouts of the animals were defined as active periods framed by inactive periods of at least 30 min [74]. Measured variables of burrow architecture (number of entrances and depth of nest) and of mothers’ activity at burrow entrances (mean number of readings per bout, mean duration of bout in seconds, and passage speed in readings per second) originated from a single burrow. Maternal activity over 36 h was analysed.

All variables were normally distributed except the number of burrow entrances (Kolmogorov–Smirnov *Z* = 1.54, *P* = 0.018), which was Poisson distributed (Kolmogorov–Smirnov *Z* = 1.03, *P* = 0.24) and therefore analysed using a general linear model (GLM). All other variables were analysed using analysis of variance (ANOVA; depth of the nest site) or multivariate analysis of variance (MANOVA; the three variables of burrow entrance activity, see above). The three models, GLM, MANOVA, and ANOVA, included treatment as a fixed factor with three levels: NP, RC, and C.

The FCMs of vole mothers and their effects on body weight were analysed with two-factorial GLMs with treatment and sampling as fixed factors.

The influences of origin (lab born vs. wild caught), age (over-wintered vs. born that year) and experience (second litter vs. > 2 litters) of mothers on all dependent variables were tested using ANOVAs or GLMs (depending on their distributions), but none of these factors had a significant impact and they are not discussed further. The number of nestlings was not significantly influenced by any treatment. For all statistical analyses, SPSS for Windows 19.x (IBM, Chicago, IL, USA) was used.

## Competing interests

All authors declare that they have no competing interests.

## Authors’ contributions

ML participated in the study design, carried out the field study, extracted FCMs, conducted statistical analyses, and drafted the manuscript. TL worked in the field and helped with statistical analysis. RP participated in the faecal sampling design and analysed the FCMs. JAE conceived of the study design. All authors corrected and improved the manuscript. All authors read and approved the final manuscript.
